# Decline in Maxillofacial Injuries during the Pandemic: The Hidden Face of COVID-19

**DOI:** 10.3390/jcm12010128

**Published:** 2022-12-24

**Authors:** Adi Kasem, Idan Redenski, Daniel Oren, Adeeb Zoabi, Samer Srouji, Fares Kablan

**Affiliations:** 1 Department of Oral and Maxillofacial Surgery, Galilee College of Dental Sciences, Galilee Medical Center, Nahariya 2210001, Israel; 2 The Azrieli Faculty of Medicine, Bar-Ilan University, Safed 1311502, Israel

**Keywords:** maxillofacial injuries, facial bone fractures, COVID-19

## Abstract

Maxillofacial injuries result from a variety of daily activities. Traffic accidents, interpersonal violence, and falls represent some of the most common etiological factors behind maxillofacial fractures. During the COVID-19 outbreak, the social distancing measures imposed by healthcare authorities aimed at abolishing the spread of the viral infection. This study aimed to evaluate the effect of social distancing measures on the incidence of maxillofacial injuries. Methods: Data were retrieved from the medical file registry at the Galilee Medical Center, Nahariya, Israel. Incidence, gender, age, etiology, and cost of hospitalization during the COVID-19 lockdown and the previous periods were retrieved. Results: A decrease in maxillofacial fractures was registered during the 2020 lockdown; younger patients had the largest share of maxillofacial traumas during this period. The midface was the most involved facial region in both periods, and a reduction of 62.3% in the cost of OMF fracture treatment was observed during the COVID-19 era. Conclusions: The occurrence, etiology, and cost of treatment of maxillofacial injuries during the COVID-19 period were different from those in the corresponding period in the pre-COVID-19 era. These results can provide a guide to help design programs for the prevention of OMF trauma.

## 1. Introduction

Maxillofacial injuries are among the most common injuries and are usually combined with fractures of facial bones. Facial bone fractures may include the zygomatic complex and the malar bones, the maxilla and the mandible, the orbital walls, the alveoli and the teeth, and the nasal and frontal sinus bones [1,2]. Different traumatic events may affect the maxillofacial region, e.g., road accidents, falls (falls from heights, falls due to systemic illness), interpersonal violence, work accidents, sport, and other injuries [3,4,5]. Israeli government organizations have been investing resources and facilities in an attempt to control and decrease those leading causes, all without improvement. In the past three years, there was an average of approximately 190,000 road accidents in Israel annually. In 2019, 81,000 people were injured, and 343 were killed there. The economic cost of road accidents in Israel is ca. 4.9 billion US dollars a year, which is about 1.3% of the national product [6].

COVID-19 is an infectious disease that was first reported in December 2019 in Wuhan, China, and has since spread globally, resulting in the ongoing coronavirus pandemic fueled by human-to-human transmission [7]. One of the essential ways to control it that has been utilized worldwide is decreasing interpersonal contact. In Israel, the first case was described in February 2020. Since March 2020, the whole country has been on lockdown to slow down the spread of the infection. In addition, there was a shutdown of public transport, road transport and trains, educational institutions, business centers, parks, and other social interaction points.

The GMC is considered a high-level trauma center localized in the north of Israel, and the Department of Maxillofacial Surgery is an essential part of this center. In our hospital, there was a significant reduction in the rate of admission of patients suffering from traumatic injuries during March and April 2020 (COVID-19 era). The aim of this study is to evaluate the occurrence of maxillofacial injuries in our hospital in the period of COVID-19 and compare it to the corresponding pre-COVID-19 era (2017, 2018, and 2019).

## 2. Materials and Methods

This retrospective study was approved by the GMC Helsinki committee. Medical files of all the maxillofacial trauma patients who were admitted to the GMC during the year 2019, as well as of the patients admitted for trauma during March and April 2020 (COVID-19 lockdown) and the corresponding periods prior to the COVID-19 lockdown, i.e., March–April for 2017 and 2018 (termed “pre-COVID periods”) were reviewed. Only patients who had sustained an injury with subsequent facial bone fractures were included in the study. Etiology of injury, age, gender, treatment, site of injury, operative time, number of plates and screws, and duration of hospitalization were all included in the analysis. The patients were divided into five groups according to the period they were admitted to the GMC trauma center. The first group included OMF fractures admitted to the hospital during the year 2019. The second, third, and fourth groups belonged to the pre-COVID-19 era and included the patients who were admitted to the hospital during March and April of each year from 2017 to 2019. The fifth group included the trauma patients admitted during March–April 2020, which corresponded to the COVID-19 lockdown.

Statistical analysis was performed using IBM SPSS statistics software version 27.0. Continuous data were described using the means and range. Categorical data were presented by frequencies and percentages. For the calculation of maxillofacial injuries incidence and odds ratios, the general population used for analysis included the entire population in the region of Acre in Western Galilee, served by the hospital during the corresponding years.

Continuous variables were analyzed using the Mann–Whitney test. Categorical variables (gender, etiological factors, trauma site, etc.) for different years were compared and analyzed using Pearson’s chi-squared test, Fisher–Freeman–Halton exact test (if expectancy < 5), and univariate logistic regression analysis (the results were reported as odds ratios (OR) and each year from the pre-COVID era was compared to the COVID-19 lockdown period in 2020); the differences were considered significant at *p* < 0.05. Two-sided *p*-values were presented unless otherwise noted.

## 3. Results

### 3.1. Incidence of OMF Admissions

**Figure 1:**
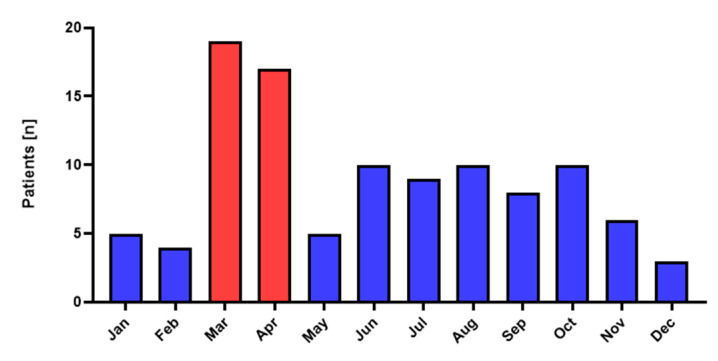
Trauma patients admitted to the Galilee Medical Center in 2019.

One hundred five maxillofacial trauma patients were admitted to the GMC emergency department in 2019. The peak in OMF fractures was registered in March and April of that year (Figure 1).

**Figure 2:**
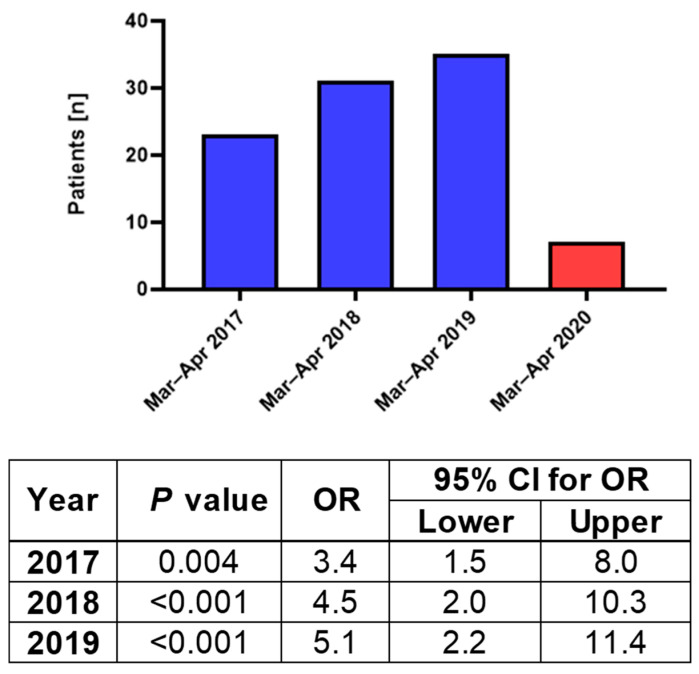
Trauma patients admitted to the Galilee Medical Center during March and April of 2017, 2018, and 2019 compared to the same period in 2020 corresponding to the COVID–19 lockdown, presented with univariate logistic regression analysis.

The patients admitted during March and April before the COVID-19 outbreak included a total of 89 maxillofacial fractures (Figure 2). The average number of monthly admissions in these periods was 12 patients, and the average age of the trauma patients admitted was 45.6 years. During the COVID-19 lockdown in 2020, seven patients were admitted to the hospital with an average age of 28.7 years upon admission.

While during the COVID-19 lockdown, the cases of facial trauma were 1.07 cases per 100,000 residents. In the previous years, 2017, 2018, and 2019, the incidence of facial trauma was 3.65, 4.86, and 5.4 cases per 100,000 residents, respectively. Logistic regression indicated significant differences compared to the 2020 COVID-19 period, being 3.4, 4.5, and 5.1 higher in 2017, 2018, and 2019, respectively (Figure 2).

**Figure 3:**
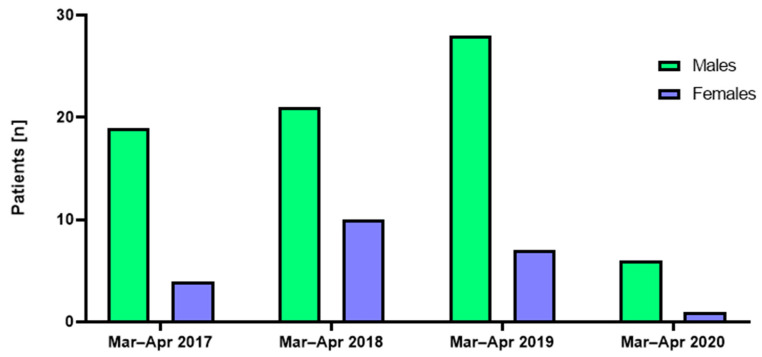
Trauma patients (males and females) admitted during March and April to the GMC trauma center in the years 2017–2020.

Both in the COVID-19 and the corresponding pre-COVID-19 periods, the admission rate of male patients was higher than that of female patients (a total of 74 male patients compared to 22 females), a ratio of 3.36/1. For males, a significantly higher occurrence of maxillofacial injuries was registered in the years 2017, 2018, and 2019, being 3.3, 3.6, and 4.7 times higher compared to the COVID-19 lockdown in 2020 (Figure 3, *p*-values = 0.011, 0.006, and 0.001, respectively). The same trend regarding the occurrence of maxillofacial injuries was registered for female patients, being 4.216, 10.27, and 7.09 higher in the years 2017, 2018, and 2019 compared to the COVID-19 lockdown in 2020 (Figure 3, *p*-values = 0.067, 0.026, and 0.202, respectively).

### 3.2. Age and Etiology

**Figure 4:**
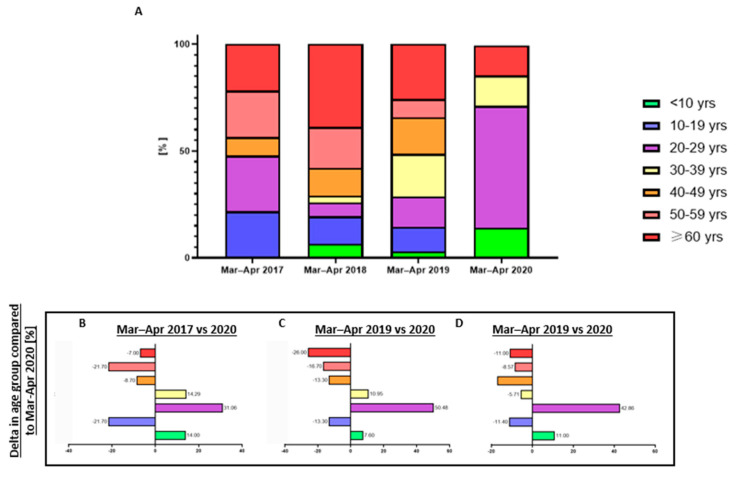
Age distribution across the trauma patients admitted during March and April to the GMC trauma center in the years 2017–2020. (**A**) Admissions as the percentage of the total admissions during March and April of each year. (**B**–**D**) Change in each age group admitted during 2017, 2018, and 2019 compared to the period corresponding to the COVID–19 lockdown.

Age ranged from 5 to 89 years, with an average age of 44 ± 23 years upon admission. In 2017, 2018, and 2019, the corresponding periods showed a clear trend, with the majority of OMF trauma patients being older than 29 years of age (52.17%, 74.19%, and 71.42%, respectively). However, while the involvement of age groups varied across the periods under investigation, one trend seemed consistent. The lockdown period (March–April 2020) showed a distinct increase in trauma patients aged 20–29 years compared to the same period in 2017, 2018, and 2019 (an increase of 31.06%, 40.46%, and 42.86% compared to 2020, respectively), reaching a total of 57.14% of the patients as described in Figure 4. While age differences were not found to be significantly different between 2017 and the year 2020, a significant difference was found via a one-sided hypothesis for 2018 and 2019 (*p*-values = 0.039 and 0.05, respectively).

**Figure 5:**
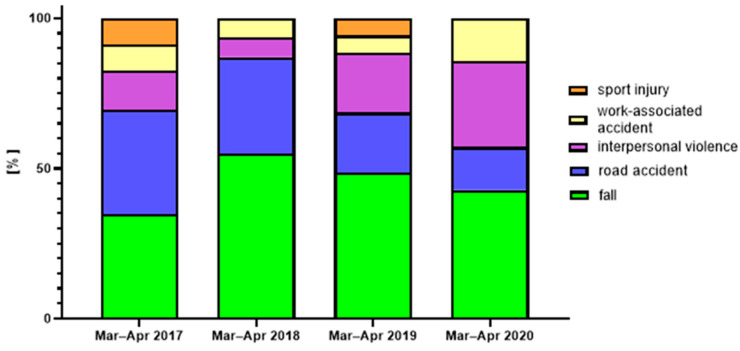
Etiology of OMF fractures admitted to the GMC trauma center during March and April in the years 2017–2020.

The leading cause of traumatic maxillofacial fractures in the patients admitted during the COVID-19 lockdown and the corresponding periods in 2017, 2018, and 2019 were falls, followed by road accidents. Together, these etiologies comprised well over 50% of the hospital admissions during March and April in 2017, 2018, 2019, and 2020 (69.56%, 87.09%, 68.57%, and 57.15%, respectively, Figure 5). Etiological factors did not show statistically significant changes before and during the COVID-19 lockdown, possibly due to the low number of facial trauma admissions during the 2020 COVID-19 lockdown.

### 3.3. Injury Site and Costs

**Figure 6:**
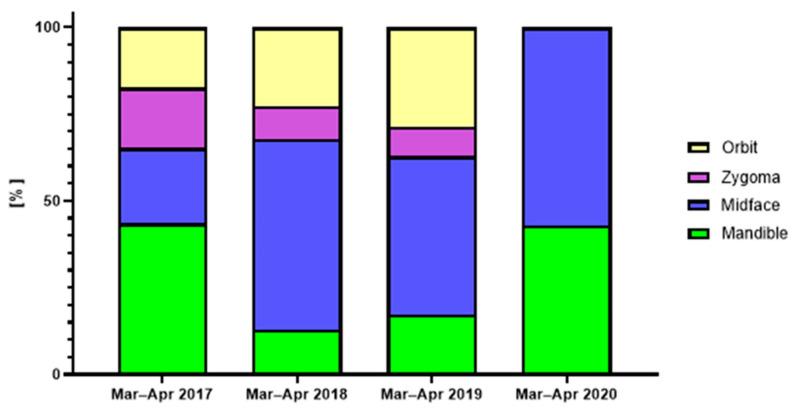
OMF fractures according to the site of injury in the patients admitted to the GMC trauma center during March and April in the years 2017–2020.

The majority of facial fractures across the periods involved both the mandible and the midface, with a cumulative occurrence of over 60% across the periods analyzed (Figure 5). Interestingly, injuries involving the upper third of the facial skeleton were not registered at all during the lockdown (Figure 6).

**Figure 7:**
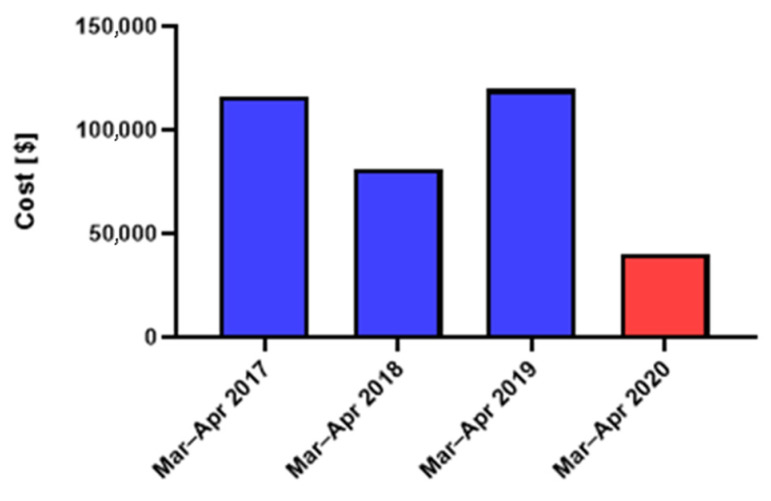
Total costs associated with maxillofacial fractures in the patients admitted during the COVID era and the corresponding periods in 2017–2019.

As for the total treatment cost during March and April in the pre-COVID-19 period as well as the corresponding COVID-19 lockdown, a reduction of 62.3% in the total cost of OMF fractures was registered (Figure 7). The average treatment cost per month during the pre-COVID-19 era was 52,874 USD, while during the COVID-19 lockdown, the treatment expenses decreased substantially, reaching as low as 19,945 USD.

## 4. Discussion

The outbreak of the COVID-19 virus was a challenge for the world community. Despite all scientific progress nowadays, social distancing and personal hygiene were the most effective tools to prevent the spread of the virus. Most countries had imposed lockdowns and social distancing, with Israel being amongst the countries that adopted a strict social distancing policy, imposing a lockdown on all amenities of life, specifically during the period between March and April 2020 [8] with the intention of slowing down the spread of the COVID-19 infection. Thus, a total shutdown of all public transportation and gathering areas was in effect and included trains, major roads closure, and shutdown of educational institutions, business centers, cultural centers, and public parks [9].

Generally, when winter ends, and spring begins, most Israeli citizens are on holiday, spending more time outdoors and having face-to-face interactions. This period usually correlates with a rise in traumatic injuries, including OMF injuries [10]. During 2019, one hundred and five patients were admitted to our department suffering from different OMF fractures, with the average number of patients per month being 8.33 patients. Specifically, during the months of March and April in the same year, the average number of patients admitted was 17.5, more than a fourfold rise in admissions of patients with OMF injuries compared to the previous months in 2019. These results were in accordance with other studies where the peak in the incidence of OMF injuries was registered in March and April [11,12].

In the pre-COVID period, during March and April, the monthly average number of patients was 14.83, with traffic accidents being the main etiological factor for OMF fractures admission. This trend corresponds with previous reports that described traffic accidents as the leading cause of OMF fractures [10,11,12]. However, during the COVID-19 lockdown, a dramatic reduction in admissions of patients with maxillofacial fractures was registered, with an average of 3.5 patients per month. The lowered admission rates were in line with reports from other healthcare centers during the pandemic [13]. The average age upon admission during the COVID-19 lockdown was 28.7 years, compared to 45.6 years during the corresponding periods in the pre-COVID era. The fact that younger patients were more involved in injuries resulting from outdoor and interpersonal activities correlates with reports regarding the perceived importance of quarantine measures amongst younger people. Among respondents on the perceived importance of social distancing measures and self-isolation guidelines, younger respondents reported lower adherence to full self-isolation periods and less compliance with social distancing measures [14]. Moreover, almost no compliance was reported among people of lower age, in children and adolescents, who were also admitted to the GMC during the pandemic [15]. Thus, the change in age distribution between the pre-COVID era and the COVID-19 outbreak corresponded to the perceived importance of safety measures amongst younger patients during these times.

A major result of the lower admissions rate during the pandemic was a reduction of 62.3% in the total cost of OMF fractures treatment at the GMC trauma center. This trend correlated with the reduced admission rate during the pandemic. However, while the cost of trauma-related therapy was reduced, it was outweighed by the cost of treatment of infected COVID-19 patients, accompanied by the dramatic reduction in elective services [16,17].

## 5. Conclusions

Treatment of trauma injuries worldwide has seen a dramatic change during the COVID-19 pandemic. These changes were also observed in Israel, with a massive reduction in admissions due to OMF fractures. Thus, the social distancing and “stay-at-home” policy had a clear positive effect on the incidence of facial injuries.
